# Prevalence of enteropathogenic viruses and molecular characterization of group A rotavirus among children with diarrhea in Dar es Salaam Tanzania

**DOI:** 10.1186/1471-2458-7-359

**Published:** 2007-12-27

**Authors:** Sabrina J Moyo, Njolstad Gro, Vainio Kirsti, Mecky I Matee, Jesse Kitundu, Samwel Y Maselle, Nina Langeland, Helge Myrmel

**Affiliations:** 1 Department of Microbiology and Immunology, School of Medicine, Muhimbili University of Health and Allied Sciences, Dar es Salaam, Tanzania; 2 Department of Microbiology and Immunology, Haukeland University Hospital, Bergen, Norway; 3 Department of Virology, Norwegian Institute of Public Health, Oslo, Norway; 4 Department of Paediatrics and Child Health, Muhimbili National Hospital, Dar es Salaam, Tanzania; 5 Institute of Medicine, University of Bergen, Bergen, Norway; 6 Department of Medicine, Haukeland University Hospital, Bergen, Norway

## Abstract

**Background:**

Different groups of viruses have been shown to be responsible for acute diarrhea among children during their first few years of life. Epidemiological knowledge of viral agents is critical for the development of effective preventive measures, including vaccines.

**Methods:**

In this study we determined the prevalence of the four major enteropathogenic viruses – rotavirus, norovirus, adenovirus and astrovirus – was determined in 270 stool samples collected from children aged 0 – 60 months who were admitted with diarrhea in four hospitals in Dar es Salaam, Tanzania, using commercially available ELISA kits. In addition, the molecular epidemiology of group A rotavirus was investigated using reverse transcriptase multiplex polymerase chain reaction (RT-PCR).

**Results:**

At least one viral agent was detected in 87/270 (32.2%) of the children. The prevalence of rotavirus, norovirus, adenovirus and astrovirus was 18.1%, 13.7%, 2.6% and 0.4%, respectively. In most cases (62.1%) of viruses were detected in children aged 7–12 months. The G and P types (VP7 and VP4 genotypes respectively) were further investigated in 49 rotavirus ELISA positive samples. G9 was the predominant G type (81.6%), followed by G1 (10.2%) and G3 (0.2%). P[8] was the predominant P type (83.7%), followed by P[6] (0.4%) and P[4] (0.2%). The following G and P types were not detected in this study population; G2, G4, G8 G10, P[9], P[10] and P[11]. The dominating G/P combination was G9P[8], accounting for 39 (90.7%) of the 43 fully characterized strains. Three (6.1%) of the 49 rotavirus strains could not be typed.

**Conclusion:**

Nearly one third of children with diarrhea admitted to hospitals in Dar es Salaam had one of the four viral agents. The predominance of rotavirus serotype G9 may have implication for rotavirus vaccination in Tanzania.

## Background

In Dar es Salaam Tanzania relatively few studies have investigated viral causes of diarrhea. These studies, which were conducted more than ten years ago, searched for a relatively narrow spectrum of viruses [1,2]. There has been only one recent study conducted in Ifakara, Tanzania which is about 500 KM away from Dar es Salaam which reported prevalence of rotavirus [3]. These studies used either latex agglutination test methods or electron microscopy, techniques which have been reported to be less sensitive and less specific than EIA [4,5]. Despite the reported predominance of rotavirus the molecular epidemiology of group A rotavirus strains in Tanzania is not known resulting in lack of important information for the introduction of a suitable rotavirus vaccine in future.

Several different groups of viruses have been shown to be responsible for the high incidence of acute viral diarrhea among children during their first few years of life [6]. These viruses include rotavirus [7], noroviruses [4], enteric adenovirus 40/41 [4] and astrovirus [8]. Among them, rotavirus is the single most important etiological agent in severe dehydrating diarrhea, causing approximately 111 million episodes of gastroenteritis, 25 million clinic visits, 2 million hospitalizations and 440,000 deaths in children aged less than five years of age each year worldwide [9]. Most human rotaviruses belong to group A [4]. The outer capsid layer of the virus consist of two proteins carrying distinct epitopes (VP7 and VP4), which are specifically determined by neutralizing antibody responses, and thus characterize serotype of rotaviruses [10]. The VP7-specific serotypes are termed G types (G stands for glycoprotein) and VP4-specific serotypes are termed P types (P stands for protease-sensitive protein). The genes coding for VP4 and VP7 proteins have been used for genotypic classification of strains. Among group A rotaviruses, 15 different G serotypes and 26 different P types have beeen identified to date and there is co-existance of different G and P combinations among rotaviruses. Initially almost all episodes were found to be caused by G1-G4 types, but gradually the occurrence of G12, G9, G5, G8 and G10 genotypes has been reported from several countries [11]. G/P combinations due to G type of VP7 gene and P VP4 gene are useful characteristics to distinguish rotaviruses. There are significant geographical differences regarding the strain distributions [12]. Studies of rotavirus strain diversity have traditionally employed VP7-specific neutralizing monoclonal antibodies in enzyme immunoassays (EIAs) to detect the common VP7 serotype in stool. However, a substantial number of strains remain nontypeable by EIA [13]. Furthermore, serologic methods to detect common VP4 serotypes are not routinely available. Currently molecular methods such as RT-PCR and probe hybridization have been developed for both VP7 and VP4 typing [13,14].

Our study aimed to determine the prevalence of four important diarrheagenic virues viz. rotavirus, norovirus, enteric adenovirus and astrovirus among children less than five years of age who had to be hospitalized due to diarrhea in Dar es Salaam, Tanzania. In addition molecular typing of Group A rotavirus strains was also performed using RT-PCR.

## Methods

### Study design and subjects

This was a prospective cross-sectional study that was conducted in Dar es Salaam city which has three districts with 4 large public hospitals and 4 big private hospitals. The four public hospitals are the largest hospitals in Dar es Salaam. Three of these (Amana, Mwananyamala, and Temeke district hospital) which are located at each district of Dar es Salaam are secondary health care facilities while one (Muhimbili National Hospital, MNH) is a tertiary health care facility. This study was conducted at MNH, Amana, Mwananyamala, and Temeke district Hospitals between December 2005 and February 2006. These hospitals handle patients with severe disease and those that need admission. Being public facilities they cover approximately 70% of the patient load and the remaining 30% is handled by the private hospitals especially for patients who are able to pay. The catchments area of these hospitals is Dar es Salaam city which has a population of around 4 million.

During the study period a total of 297 children who were hospitalized owing to diarrhea were enrolled in this study. Enrolment was subject to obtaining an informed verbal consent from the parent or guardian who accompanied the child. Children whose parents/guardians did not consent and those whose age could not be ascertained were excluded from the study. Seventeen of these children were excluded from the study due to lack of consent or uncertainty of age while 10 were excluded due to improperly collected specimen.

### Demographic and clinical information

A standard structured questionnaire was used to obtain the information regarding the age, sex, duration, frequency of diarrhea and consistency of the stool (as watery, mucoid, or bloody) for each case. Diarrhea was defined, according to WHO guidelines [15], as the occurrence of three or more, loose, liquid, or watery stools within 24 hour period. The guidelines define three forms of diarrhea viz. i) acute watery diarrhea for diarrhea that began acutely and lasted less than 14 days, ii) dysentery defined as mucoid bloody stool associated with anorexia, abdominal cramps, and tenesmus and iii) persistent diarrhea defined as diarrhea with a duration of 14 or more days without remission in between. Information regarding the use of antibiotics prior to hospitalization was also sought.

### Weight measurements

Infants under two years of age were weighed using a 25 kg Salter hanging scales (CMS Weighing equipment, High Holborn, London, United Kingdom). Children over two years of age were weighed while standing on scales that were calibrated before each session. Weight of children was recorded to the nearest kilogram. Weight-for age Z-scores were calculated using EPI Info (USD, Inc., Stone Mountain, GA). According to WHO criteria [16] children were considered to be undernourished if the Z-scores were less than -2SD.

### Specimen collection and transportation

Stool specimens were collected using wide mouthed sterile plastic containers and a portion of specimen was stored in 1.8 m plastic nunc tubes (Nalge Nunc International, NY, USA) at -20°C until the time for analysis. All the specimens were subjected to one cycle of thawing and freezing.

### Laboratory procedures

#### Detection of rotavirus, norovirus, adenovirus and astrovirus

The presence of four enteric viruses namely Rotavirus, norovirus, adenovirus and astrovirus antigens was investigated using the suspension of stool samples by commercial immunoassays IDEIA Rotavirus, IDEIA Adenovirus, and IDEIA Astrovirus and IDEIA Norovirus (Dako Ltd., Ely, United Kingdom) according to the manufacturer's instructions.

### Molecular typing of rotavirus

#### RNA extraction and Reverse Transcription (RT)

Rotavirus antigen-positive stool samples were mixed 1:10 with Eagles Minimum Essential Medium, and clarified by low-speed centrifugation. Double-stranded RNA was extracted from 140 μl supernatant using the Magna Pure LC Total Nucleic Acid Isolation Kit (Roche Applied Science). RNA was additionally extracted from a subset of samples using the QIAamp Viral RNA Mini Kit (Qiagen/Westburg, Leusden, and the Netherlands). Both kits were used according to the manufacturer's instructions. 40 μl of RNA extract was used as template for RT with random primers.

#### Rotavirus genotyping using multiplex PCR

Rotavirus G an P genotyping was performed using semi-nested type specific multiplex PCR's that could detect seven G-types (G1, G2, G3, G4, G8, G9, G10) and six P-types P[4], P[6], P[8], P[9], P[10] and P[11], respectively [17]. The P-typing was performed using modified first round primers according to rotavirus detection and typing protocol provided by Dr J. Gray, HPA, UK. Briefly, the first round VP4 and VP7 consensus PCR's were carried out with 5 μl of cDNA in 50 μl of each of the VP4 and VP7 reagent mixtures, respectively. The second round VP4 and VP7 multiplex PCR's were carried out with 2 ul of first round VP4 and VP7 amplicons in 50 μl of each of the second round VP4 and VP7 reagent mixtures, respectively. The multiplex PCR mixtures contained 10x buffer II without MgCl_2_(Invitrogen), MgCl_2 _(2 mM and 2.5 mM respectivel *y*), deoxynucleoside triphosphates (0.2 mM), primers (20 pmol), and *Taq *DNA polymerase (1U) (Invitrogen). PCR was performed at 94°C for 2 min, followed by 35 cycles at 94°C for 1 min, 50°C and 52°C for 1 min, and 72°C for 1 min and a final extension at 72°C for 7 min, and then the mixture was held at 15°C. PCR products were subjected to electrophoresis on a 2% agarose gel, stained with ethidium bromide, and observed under ultraviolet light. The different G and P types were analyzed by comparing the size of the second round PCR-products with PCR-products of known rotavirus strains. The untypeable rotavirus strains were further analyzed using a single-round rotavirus VP6 specific PCR following rotavirus detection and typing protocol provided by Dr J. Gray, HPA, UK. Negative and positive controls were used in all PCR assays.

#### Data management

The Statistical Package for the Social Sciences (SPSS for WINDOWS version 10.0) was used for statistical analysis. Weight-for age z scores were calculated using EPI Info (USD, Inc., Stone Mountain, GA). Assuming the data follows a normal distribution, comparison of proportions and statistical significance were tested by using the Chi-square test. A p value less than 0.05 was considered statistically significant

#### Ethical considerations

Ethical and research clearance was obtained from the Higher Degree Research and Publication Committee, Muhimbili University College of Health Sciences, Dar es Salaam. Permission was sought from the respective hospital authorities. Informed verbal consent was obtained from parents/caretaker of the child before enrolment into the study. Children were treated according to IMCI guidelines [15].

## Results

### Prevalence of rotavirus, norovirus, adenovirus and astrovirus

Among the 270 stool specimens collected from children with diarrhea, 87 (32.2%) contained at least one of the four viruses. Table 1 shows the frequency of each individual viral agent as follows: rotavirus 49 (18.1%), norovirus 37(13.7%), enteric adenovirus 7(2.6%) and astrovirus 1 (0.4%). Using commercial EIA's kit, multiple viral infections were found in seven out of the 87 positive samples (8.0%). Six were co-infections of rotavirus and norovirus and one sample contained rotavirus and adenovirus.

**Table 1:** Prevalence of four enteric viruses among underfives with diarrhea in Dar es Salaam Tanzania.

Enteric virus	n = 270	%
No of children with at least one virus	87	32.2
Rotavirus	49	18.1
Norovirus	37	13.7
Adenovirus	7	2.6
Astrovirus	1	0.4
Dual infection	7	2.6

### Association between age group and viral enteric pathogens isolated

As shown in Table 2, only one virus (norovirus) was detected in the age group of 0–2 months, the prevalence of viruses was low in infants aged 0–6 months, peaked in infants aged 7–12 months and declined again among children of 13–16 months of age (p >0.05).

**Table 2:** Association between age group and viral enteric pathogens isolated among under fives with diarrhea in Dar es Salaam

	Total	Rotavirus	Norovirus	Adenovirus	Astrovirus
*Age group in months*					
0–2	4	0	1 (2.8)	0	0
3–6	46	13 (26.0)	8(22.2)	0	0
7–12	133	25 (52.0.)	20(54.2)	6(85.7)	1(100)
13–24	63	9(18.0)	7 (19.4)	1(14.3)	0
25–60	24	2 (4.0)	1(2.8)	0	0
**Total**	270	49	37	7	1

Percentages in brackets

### Rotavirus genotyping

Rotavirus was detected in 49 out of 270 (18.1%) samples tested. Of the 49 rotavirus ELISA positive samples, 43 (87.7%) were fully G and P genotyped by RT-PCR, 3 (6.0%) were partially genotyped, while 3 (6.0%) could not be genotyped. In total 46 samples were G typeable, and the dominant G type was G9 40 (81.6%), followed by G1 (10.2%), and G3 (2.0%) (Table 3). The genotypes G2, G4, G8 and G10 were not detected in this study. As shown in Table 4, 44 samples were P typeable. P[8] predominated accounting for 41 (83.7%) of the 44 P typeable strains. Only two P[6] strains and one P[4] strain was detected, whereas P[9], P[10] and P[11] strains were not detected. The most prevalent P/G combination was G9P[8], accounting for 39/43 (90.7%) of the fully characterized strains. The two P[6] isolates were G1P[6], where as one P[4] was untypeable.

**Table 3:** Prevalence of G genotype of Group A rotavirus detected among underfive children with diarrhea in Dar es Salaam Tanzania

*G genotype*	n = 49
G9	40(81.6%)
G1	5 (10.2%)
G3	1(2.0%)
G2,G4,G8 and G10	0(0.0%)
G untypable	3 (6.1%)

**Table 4:** Prevalence of P genotype of Group A rotavirus detected among underfive children with diarrhea in Dar es Salaam Tanzania

*P genotype*	n = 49
P[8]	41 (83.7%)
P[6]	2(4.1%)
P[4]	1(2.0%)
P[6], P[9], P[10] and P[11]	0(0.0%)
P untypable	5 (10.2)

## Discussion

The present study was conducted during the dry season of the year and showed that nearly one-third (31.9%) of diarrhea among under fives in Dar es Salaam is due to the four major viruses causing diarrhea in children, namely rotavirus, norovirus, adenovirus and astrovirus. Of the four viruses, rotavirus was by far the most frequently isolated, accounting for 57.5% of all the viruses and followed by norovirus that accounted for 42.5% of the viruses. Overall rotavirus accounted for 18.5% of all cases of diarrhea which is lower than other findings in Tanzania which reported rotavirus to account for up to 43% of diarrhea cases [2,3]. The difference between these two studies could be explained, at least in part, due to seasonal differences and by the use in the previous studies of the less specific agglutination tests which may lead to false positive results compared to ELISA [18].

The frequency of detection of norovirus found in this study (13.7%) is in keeping with numerous other studies conducted in both developing and developed countries that have reported norovirus to be responsible for between 10 and 15% of all cases of gastroenteritis [4]. This is one of the very few reports on norovirus gastroenteritis in developing countries [19-22], therefore further studies are needed to ascertain the disease burden associated with this virus in developing countries.

We found a relatively low rate of enteric adenovirus (2.6%) which is in agreement with a previous report of 2.7% in Dar es Salaam[1] suggesting that adenoviruses play relatively little role in causing diarrhea among children in Tanzania.

We detected only one astrovirus (0.4%) in this study group. This is the first report to examine the epidemiology of astrovirus in patients with gastroenteritis in Tanzania, and our finding suggests that the virus account for only a minority of diarrhea cases.

Using commercial EIA kit, we found dual viral infections among seven (8.0%) samples, all of which were combinations of rotavirus and one of the other viruses. E.Roman *et al *2003 [23], reported high incidence of dual infections during autumn than other seasons of the year this can not be concluded in this study since it was conducted in only one season of the year. Despite using standard multiplex PCR typing, no mixed rotavirus infections were detected in this study. In other studies, mixed rotavirus infections have been identified to range from less than 1% to 44.6 % of the samples [24-30]. Differences found between this study and other studies may be explained, at least in part, due to geographical differences.

Overall the prevalence of viral diarrhea was low in infants aged 0–6 months, peaked in infants aged 7–12 months and declined again among children of 13–16 months of age. The relatively low prevalence of viruses among older children could be partly due to immunity acquired through previous exposures.

This first report on molecular epidemiology of rotavirus strains in Tanzania show unexpectedly high proportion of G9 serotype (81.6%). The high proportion of G9 in this study is in contrast with findings of other developing countries such as Kenya, Malawi and Ghana, where the highest reported prevalence of G9 was 47.1% [31-38]. The complete absence of serotypes G2 and G4 in this study group is in marked contrast to the global situation, where these strains are often the most common [12]. This variation of strain distribution does underline the importance of active rotavirus strain surveillance in a variety of geographical settings. Our findings may have significant implications for rotavirus vaccine introduction in Tanzania since the two vaccines that are in use namely RotaRix^® ^and RotaTeq^® ^contains serotype G1 and serotype G1 to G4, respectively [10]. Notably, the globally most prevalent P[8] genotype was detected for VP4 in 93.7% of the P-typed strains and the dominating G/P combination was G9 P[8] (90.7%). It is important to mention that this study dealt with strains isolated from only one city in Tanzania (in and around Dar es Salaam) and the period of surveillance was limited to a period of 3-months. It will be important to continue strain characterization in Dar es Salaam and initiate studies in other regions of Tanzania in order to have a comprehensive picture of strain distribution in the country. It is equally important to maintain continuous surveillance for rotavirus strains in order to monitor changes over a time period.

## Conclusion

This study demonstrated that about one third of childhood diarrhea in Dar es Salaam is due to one of the four major enteropathogenic viruses and rotavirus genotype G9 predominated. The predominance of this emerging strain has significant implications for introduction of rotavirus vaccine in Tanzania. Our study indicates that geographical P-G type adjustment in the formulation of next generation multivalent vaccine would be necessary.

## Competing interests

The author(s) declare that they have no competing interests.

## Authors' contributions

SJM was the principal investigator, who conceived and designed study and was responsible for collection of specimens and clinical information as well as data analysis. Laboratory investigations were performed by SJM, NG and KV. SYM, MIM, JK, NL and HM assisted in the development of the research proposal, data analysis and preparation of the manuscript. All authors have read and approved the final manuscript.

## Pre-publication history

The pre-publication history for this paper can be accessed here:


